# Glymphatic System a Window on TBI Pathophysiology: A Systematic Review

**DOI:** 10.3390/ijms23169138

**Published:** 2022-08-15

**Authors:** Michela Ferrara, Giuseppe Bertozzi, Gianpietro Volonnino, Nicola Di Fazio, Paola Frati, Luigi Cipolloni, Raffaele La Russa, Vittorio Fineschi

**Affiliations:** 1 Department of Anatomical, Histological, Forensic and Orthopaedic Sciences, Sapienza University of Rome, 00161 Rome, Italy; 2 Department of Clinical and Experimental Medicine, Section of Legal Medicine, University of Foggia, 71122 Foggia, Italy

**Keywords:** glymphatic system, TBI, AQP4

## Abstract

Background: In recent years, the attention of the scientific world has focused on a clearance system of brain waste metabolites, called the glymphatic system, based on its similarity to the lymphatic system in peripheral tissue and the relevant role of the AQP4 glial channels and described for the first time in 2012. Consequently, numerous studies focused on its role in organ damage in cases of neuropathologies, including TBI. Methods: To evaluate the role that the glymphatic system has in the pathogenesis of TBI, on 23 March 2022, a systematic review of the literature according to PRISMA guidelines was carried out using the SCOPUS and Medline (via PubMed) databases, resulting in 12 articles after the selection process. Discussion and conclusion: The present review demonstrated that an alteration of AQP4 is associated with the accumulation of substances S100b, GFAP, and NSE, known markers of TBI in the forensic field. In addition, the alteration of the functionality of AQP4 favors edema, which, as already described, constitutes alterations of secondary brain injuries. Moreover, specific areas of the brain were demonstrated to be prone to alterations of the glymphatic pathway, suggesting their involvement in post-TBI damage. Therefore, further studies are mandatory. In this regard, a study protocol on cadavers is also proposed, based on the analyzed evidence.

## 1. Introduction

In recent years, the attention of the scientific world has focused on a clearance system of brain waste metabolites described for the first time in 2012 [1] called the glymphatic system, which connects the cerebrospinal fluid (CSF) and the interstitial fluid (ISF). This system represents a potential target for the treatment of some neurodegenerative diseases such as Alzheimer’s disease, Parkinson’s disease, amyotrophic lateral sclerosis, some psychiatric disorders, and glaucoma [2,3,4,5,6,7,8], and its impairment has been shown to play a relevant role in alterations related to brain trauma [9,10]. Historically, it has always been thought that the central nervous system was a closed system whose interaction with the rest of the organism was strictly controlled by the BBB. Therefore, in 2012, Iliff et al. introduced, for the first time, a clearance system of waste molecules from the brain called the glymphatic system [1], so named as it consists mainly of glial cells forming perivascular tunnels, analogously to the lymphatic system. Subsequent studies have then confirmed that cerebrospinal fluid and interstitial fluid are continuously exchanged through the glymphatic system, a highly organized transport pathway that consists of three main moments: periarterial CSF influx, convective/diffusion flow in the interstitial space, and ISF efflux [11].

Traumatic brain injury (TBI), on the other hand, designates physical damage to brain tissue resulting from a bump, a jolt, or a blow to the head for a direct impact or rapid acceleration–deceleration [12,13,14,15,16]. It represents a leading cause of death, long-term impairment, disability, and poor quality of life worldwide, affecting millions of people throughout the world every year [17].

According to the Centers for Disease Control and Prevention (CDC), there were about 223,000 hospitalizations related to TBI in 2019 in the United States and about 64,300 deaths in 2020 [18].

TBI is most frequently found following road accidents, falls, assaults, and sports activities [19,20,21,22]. In soldiers, it often occurs as a result of explosions [23].

Depending on the severity of the injury, the site, and the pre-traumatic history, TBI outcomes and severity can vary widely, from a temporary loss of consciousness to death or permanent disability, including motor, sensory, language, and cognitive impairments.

According to the Glasgow coma scale (GCS), TBI can be classified as mild (GCS 13-15), moderate (CG 9–13), or severe (CG 3–8). In mild trauma, the patient generally recovers his neurological function, while a severe injury causes a comatose state [24].

With regard to pathophysiology, it is possible to differentiate between a Primary Brain Injury and a Secondary Brain Injury. A Primary Brain Injury can be due: (a) to the direct impact of a mechanical force, resulting in focal damage, characterized by fractures, cerebral hemorrhages, and focal neuronal necrosis or (b) to rapid acceleration and deceleration forces that determine the stretching of the brain tissue, with related diffuse axonal damage, mostly represented at the level of the brain stem and corpus callosum, which can persist for months after the trauma. A Secondary Brain Injury, due to biochemical and cellular alterations secondary to the primary insult, is linked to numerous factors, including lipid peroxidation, mitochondrial dysfunction, oxidative stress, excitotoxicity, neuroinflammation, and axonal degeneration [25,26,27,28,29]. Given the complexity of its pathophysiology, TBI are always the subject of detailed study by the forensic sciences [30,31,32,33,34,35,36,37,38,39].

Thus, the purpose of this review is to evaluate the role that the glymphatic system has in the pathogenesis of TBI, the understanding of which could be of crucial importance for improving the outcome of subjects suffering from TBI in the future. In addition, a pilot study on a post-mortem human model is proposed, according to the evidence from the literature.

## 2. Methods

On 23 March 2022, a systematic review of the literature according to the Preferred Reporting Items for Systematic Reviews and Meta-Analyses (PRISMA) guidelines [40] was carried out using the SCOPUS and Medline (via PubMed) databases, using the following search strings: (glymphatic system) AND (traumatic brain injury), (glymphatic system) AND (TBI), and (glymphatic system) AND (trauma).

### 2.1. Inclusion and Exclusion Criteria

The inclusion criteria were: case report, original article, short survey, article in English, animal study, and human study. The exclusion criteria were: articles not in English, abstract, editorial, review, erratum, book chapter, note, and conference paper. All articles focused on other topics were excluded.

### 2.2. Quality Assessment and Critical Appraisal

M.F. and G.B. evaluated the entire texts of the articles, independently. The articles on which there was a disagreement were discussed with the senior investigator, V.F., for the final decision.

### 2.3. Risk of Bias

The main risk was linked to the keyword selected for the search strings. Therefore, the Kappa interobserver variability coefficient showed “almost perfect agreement” (0.87) [41].

### 2.4. Characteristics of Eligible Studies

A total of 251 articles were identified. 132 articles duplicates were removed, and 73 articles did not meet the inclusion criteria. After the selection process, 12 articles were included in the present systematic review.

## 3. Results and Discussion

**Figure 1:**
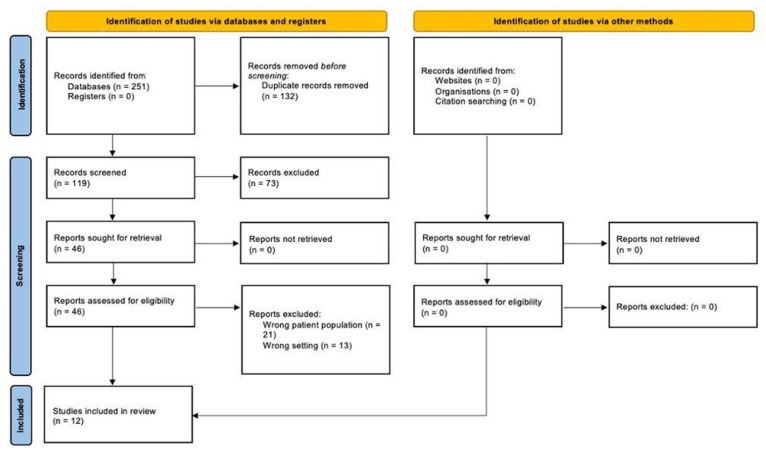
PRISMA flow diagram of this systematic review.

Of the 251 articles found, 12 articles met the inclusion criteria (Figure 1).

The selected articles underwent a critical review, detailed in Table 1.

The main features of each article included in this review are summarized in Table 2. Analyzing the results of this review, it emerges that eight were conducted on mice and four on human subjects. Of the latter, only one involved a post-mortem investigation.

As regards the experimental mice TBI models included in this review, in one case, a subarachnoid hemorrhage was produced using the endovascular perforation method; in three cases, a craniotomy was performed to produce a Controlled Cortical Impact, and in the remaining four studies, no cranial windows (“Hit & Run” model) or skull lacerations were described.

### 3.1. Alteration of Glymphatic Pathways after TBI

Christensen et al. [43] studied glymphatic clearance using contrast MRI in mouse models after a mild TBI in vivo. They found an increased lymphatic influx in the olfactory bulb and limbic structures (amygdala, hippocampus, and hypothalamus), while the efflux appeared reduced.

Li et al. [45] also used contrast MRI for the same purpose. Their results, however, showed a global alteration of the glymphatic system involving both the inflow and outflow pathways, more pronounced in the cortex, hypothalamus, and thalamus than in the cerebellum and olfactory bulb.

Piantino et al. [51] found an increased volume of perivascular spaces after mTBI, which could be involved in an impairment of waste product clearance.

Ren et al. [52] studied AQP4 expression and localization both in mild and moderate TBI. They found an increase in this channel expression in association with their depolarization. These alterations were correlated to the proportions of reactive astrogliosis; therefore, the authors suggest that targeting reactive gliosis may represent a therapeutic strategy to normalize AQP4 expression and improve the clearance of interstitial wastes after moderate or severe TBI.

Opel et al. [50] conducted a study aimed at evaluating the relationship between TBI and enlargement of the perivascular spaces by visualizing them on MRI. This work found that the enlargement of the perivascular spaces is independently related to a reduction in sleep time. However, this correlation was more significant in subjects who had undergone a TBI than in the group with a negative history of head trauma events. This suggests that there may be a correlation between the TBI and the enlargement of the Virchow–Robin spaces, which could represent a marker for reduced clearance of the glymphatic system.

### 3.2. Cellular Factors Released after TBI

According to Iliff et al. [44], the deletion of the aquaporin 4 gene causes an increase in the levels of the Tau protein after TBI. This promotes the intracellular aggregation of the protein, with concurrent axonal degeneration, neuroinflammation, and worsening of cognitive impairments.

Plog et al. [55] evaluated experimentally the clearance of TBI-specific markers such as S100b, GFAP, and NSE. While a significant increase in the serum levels of these markers was observed in wild-type mice after TBI, the serum levels of these markers in AQP4 knockout mice were similar to those in non-trauma wild-type mice, suggesting that the alteration of this channel reduces the clearance of TBI biomarkers. The same result was also obtained by altering the glymphatic function through: the administration of acetazolamide, which inhibits the production of cerebrospinal fluid at the level of the choroid plexuses; the execution of a cisternotomy of the cisterna magna (which is involved in the drainage of the cerebrospinal fluid); and through sleep deprivation.

Liu et al. [47] investigated the role of AQP 4 in immediate brain damage within 72 h of trauma (early brain injury) after subarachnoid hemorrhage using MRI with contrast injected into the cisterna magna. While in wild-type mice, the contrast was totally eliminated 3 h after its injection, in AQP4 knockout mice, its elimination was markedly reduced, suggesting the role of AQP4 in ISF transport.

These data agree with the study by Lindblad et al. [54], according to which, intracranial NSE would be eliminated by a path other than BBB, suggesting that the glymphatic system may be involved in the clearance of this biomarker.

In light of these results, an attractive therapeutic approach could be the restoration of the polarization of the AQP4 channels. Zhang et al. [48] found that omega-3 intake could represent a potential therapeutic approach for astroglial alterations following a TBI, as administering fish oil for a period of two months before determining a TBI significantly improves the neuronal function in mouse models, favors the clearance of radiolabeled tracers, and prevents the accumulation of Aß by partially restoring the expression and depolarization of AQP4 impaired by TBI.

Conversely, Liu et al. [46] showed that AQP4 knockout promotes the clearance of β-amyloid and its precursor (amyloid precursor protein—APP) after head trauma, which reduces the brain cytokine levels, improving neuroinflammation and inducing the upregulation of occludin and ZO-1 at the BBB level, preserving its integrity.

### 3.3. Post-Mortem Investigations

Olczak et al. [53] carried out a study on corpses in which head injury was the suspected cause of death to investigate the relevance of the Tau protein as a marker for TBI in post-mortem investigations. They collected blood and CSF within a day after death and frontal brain samples during forensic autopsy. Brain samples were studied using standard hematoxylin–eosin staining and immunohistochemistry (anti-human Tau antibody, CD68—anti-human macrosialin antibody, CD34—anti-human endothelial cells, and GFAP—anti glial fibrillary acid protein). They found elevated Tau protein levels in the serum and CSF of individuals dead from TBI compared to the control group. In addition, immunohistochemical analysis in the TBI group revealed astroglia damage, endothelial alteration with perivascular erythrocytes infiltration, microglia activation, and macrophages infiltration. In the light of these results, the authors suggest that elevated Tau protein serum and CSF levels could be due to different mechanisms, BBB alterations, via glymphatic pathways, or via intramacrophage transport and that are useful TBI markers for forensic purposes.

### 3.4. Neurological and Behavioral Alterations

Preclinical studies conducted on mouse models have shown that TBI is able to determine neurological and behavioral alterations such as alterations in consciousness, cognitive functions, coordination, and spontaneous locomotor activity [43,44,46,48,52]. These pathological expressions are probably related to the accumulation of neurotoxic substances due to the altered lymphatic influx/outflow related to the trauma and the consequent neuroinflammation.

As a result of the study of the literature, it emerged that the flow between CSF and ISF, through the glymphatic system, can be described in detail as follows. In the subarachnoid space, CSF is pushed into the Virchow–Robin spaces by a combination of arterial pulsatility (determined by the physiological cardiac cycle) and pressure gradients between the cerebrospinal fluid and the free fibrous matrix of the perivascular space [55]. The subsequent transport of cerebrospinal fluid into the neuropil of the dense brain parenchyma is gathered by the AQP4 water channels, whose highly polarized expression was identified on end feet rather than the soma and practically absent on brain endothelial cells [56,57]. In the interstitium, CSF pushes ISF through a polarized net directed towards the perineuronal and perivascular venous spaces. The movement of fluids occurs exclusively by diffusion in the extracellular space, with a convective flow component present only in the perivascular spaces.

ISF is then collected in the perivenous space from where it flows from the brain towards the cervical lymphatic system. Alternatively, cerebrospinal fluid can drain along the perineural sheaths of the cranial and spinal nerves, meningeal lymphatics, and arachnoid granulations. A primary CSF exit site known in both mice and humans is along the olfactory nerve, passing through the cribrose membrane to the nasal mucosa, reaching the cervical lymphatics.

This highly polarized system of convective fluid flows with a rapid exchange between cerebrospinal fluid and interstitial fluid has been called the glymphatic system based on its similarity to the lymphatic system in peripheral tissue and the relevant role of the AQP4 glial channels.

These channels are essential for the functioning of this system, which appears to play a key role in some pathological brain conditions, including Alzheimer’s disease. Iliff et al. showed, indeed, that the knockout of the gene for AQP4 in mouse models leads to an impairment of this system, with a consequent clearance reduction of the interstitial radiolabeled ß-amyloid [1].

Furthermore, numerous studies show a correlation between TBI or repetitive TBI and dementia or progressive neurodegeneration, and this is due to the accumulation of proteins such as ß-amyloid and Tau, which persist for months after a TBI [58,59]. Moreover, as demonstrated by the present literature review, an alteration of AQP4 is associated with the accumulation of other substances (S100b, GFAP, and NSE), known markers of TBI in the forensic field [44,53,54,55,60,61]. In addition, the alteration of the functionality of AQP4 favors edema, which, as already described, constitutes an alteration of secondary brain injury [56].

Animals lacking astrocytic AQP4 showed a lower CSF flow and decreased solute clearance in the parenchymal interstitium.

AQP4 is a transmembrane channel protein with a molecular weight of about 30 kDa and a tetrameric structure, responsible for the transport of water through the central nervous system, influencing both the cell volume and the space of the extracellular space. Water homeostasis affects the ionic concentration in the extracellular fluid, which, in turn, affects the concentrations of substances within neurons, including neuroactive compounds.

Moreover, and even more intuitively, these transport channels could be directly implicated in the conditions of the variation of the components of cerebral water: edema and hydrocephalus.

The latter has been associated with an upregulation of AQP4, so much so that the modulation of AQP4 has been proposed as a possible therapy, increasing the liquidation of the cerebrospinal fluid or intervening in the production of cerebrospinal fluid at the onset of the disease.

Another relevant pathology is ischemic stroke, in which AQP4 is overexpressed at the site of the injury.

Furthermore, a reduction was observed between sleep deprivation and the reduced lymphatic clearance of interstitial waste, including soluble Aβ. In addition, sleep deprivation in mouse models is associated with the depolarization of AQP4 channels.

At the astrocytic level, AQP4 is co-expressed with glutamate receptors (GLT), with which it forms complexes. A reduced absorption of this neurotransmitter was found in knockout mice for the AQP4 gene. Therefore, altering AQP4 could be implicated in the regulation of neuronal plasticity and memory, affecting the brain concentration of GLT.

As far as the areas of the brain prone to alterations of the glymphatic pathway are concerned, the imaging studies have shown: an enlargement of the perivascular spaces, as well as of the hypothalamus, thalamus, hippocampus, amygdala, olfactory bulb, and cortex, suggesting their involvement in post-TBI damage [43,50,51].

Therefore, further studies to investigate changes in the glymphatic system after TBI are needed.

In this framework, it must be considered that, although experimental models (controlled cortical impact and fluid percussion injury) provide great support in understanding the pathophysiology of TBI and its repercussions on the glymphatic pathway, they require the creation of a cranial window or fracture of the skull. This makes the animal model different from the patient with TBI, as most patients with TBI report brain injuries following a contusion, and a TBI is rarely associated with open head injuries. In addition, surgical craniectomy has been observed to impair the upregulation of cerebral AQP4 observed after closed head injury [62], and anesthetics such as isoflurane can promote tau phosphorylation and neurofibrillary pathology [63]. The TBI “Hit & Run” experimental model produces a cranial contusion whose extent can be modulated and does not require the opening of the case and employs reduced anesthesia time, thus reducing the confounding factors [52].

However, in consideration of the unethicality of a histological study on living, a study on a post-mortem human model would be desirable. A study protocol, formulated on the evidence obtained in light of this literature review, should include, at a minimum, the following steps:

1. Sampling and fixation in formolic solution of the brain as a whole in a study group (subjects who died of head trauma) and a control group (subjects who died of other causes, excluding subjects suffering from encephalopathies);
2. Sampling of the following brain areas: periventricular spaces, as well as of the hypothalamus, thalamus, hippocampus, amygdala, olfactory bulb, and cortex;
3. Standard H&E and immunohistochemical staining with anti-AQP4 antibodies, anti-S100b, anti-GFAP, anti-NSE, anti-Tau, anti-CD68, anti-CD34, anti-GFAP, and anti-ß-amyloid;
4. Blood and CSF collection for MAPT evaluation using ELISA (enzyme-linked immunosorbent assay) [50].

## 4. Conclusions

Initially imagined as a closed system, it is now established that the brain has its own lymphatic system, through which it eliminates waste substances. The existence of this functional structure is particularly relevant in organ damage related to numerous CNS pathologies, including TBI. The present review highlighted that there are still few studies conducted on human models because of the unethicality linked to the sampling of nervous tissue in vivo for research purposes. Therefore, further studies are mandatory on cadavers in order to better characterize the correlation between glymphatic system impairment and TBI, define new therapeutic targets, and improve the outcomes of the affected patients.

## Figures and Tables

**Table 1:** Critical review of the selected manuscript [42].

	Christensen et al. [43]	Iliff et al. [44]	Li et al. [45]	Liu et al. [46]	Liu et al. [47]	Zhang et al. [48]	Plog et al. [49]	Opel et al. [50]	Piantino et al. [51]	Ren et al. [52]	Olczak et al. [53]	Lindblad et al. [54]
Explicit study question	Yes	Yes	Yes	Yes	Yes	Yes	Yes	Yes	Yes	Yes	Yes	Yes
Innovative or relevant	Yes	Yes	Yes	Yes	Yes	Yes	Yes	Yes	Yes	Yes	Yes	Yes
Review of the literature	Yes	Yes	Yes	Yes	Yes	Yes	Yes	Yes	Yes	Yes	Yes	Yes
Potential to advance scientific knowledge	Yes	Yes	Yes	Yes	Yes	Yes	Yes	Yes	Yes	Yes	Yes	Yes
Clearly written and well-organized	Yes	Yes	Yes	Yes	Yes	Yes	Yes	Yes	Yes	Yes	Yes	Yes
Eligibility criteria for inclusion and exclusion of studied subjects clearly stated	Yes	Yes	Yes	Yes	Yes	Yes	Yes	Yes	Yes	Yes	Yes	Yes
Ethics approval and/or informed consent obtained	Yes	Yes	Yes	Yes	Yes	Yes	Yes	Yes	Yes	Yes	No	Yes
Conflicts of interests declared	Yes	Not specified	Yes	Yes	Yes	Yes	Yes	Yes	Yes	Yes	Yes	Yes
Sample size	The exact number was not specified	The exact number was not specified	Yes	Yes	Yes	The exact number was not specified	The exact number was not specified	Yes	Yes	The exact number was not specified	Yes	The exact number was not specified
Randomization process and/or blinding techniques described	Yes	Yes	Yes	Yes	Yes	Yes	Yes	Yes	Yes	Yes	No	Yes
Statistical analysis and/or softwares used	Yes	Yes	Yes	Yes	Yes	Yes	Yes	Yes	Yes	Yes	Yes	Yes
Bias and limitation mentioned	Yes	Not specified	Yes	Yes	Not specified	Yes	Yes	Yes	Yes	Not specified	Not specified	Yes

**Table 2:** Results of the systematic review.

Reference	Research Subjects	Aim of the Study	TBI Model and Severity	Findings and Observed Neurological/Behavioral Investigations.
Christensen et al. [43]	Mice	To examine glymphatic function after repetitive mild TBI (RmTBI) evaluating signal intensity of Gadovist, previously injected in the cisterna magna, using in vivo MRI.	Three RmTBI using a lateral impact device (50 g weight propelled at an average speed of 9.02 ± 0.18 m/s), without eye or head lacerations.	In hypothalamus, hippocampus, amygdala, and olfactory bulb after RmTBI the glymphatic influx was increased while the efflux was slower. Behavioral test used: - Time-to-right test: to measure the time between injury impact and when the animal regained muscle tone; animals in the RmTBI group required significantly more time to right themselves following injury compared to the sham animals, demonstrating an increased loss of consciousness. - Beam walking test: to assess balance and motor coordination capabilities; RmTBI animals experienced significantly more hindleg footslips in comparison to the sham animals, suggesting impaired balance and motor coordination. The authors hypothesize that inefficient waste removal results in an accumulation of neurotoxic waste within the CNS, resulting in neurological deficits observed in post-concussion symptoms. The combined increase in glymphatic influx and evidently reduced outflow in the olfactory bulb and limbic structures could promote glucose toxicity in these regions and neuroinflammatory response, altering the post-injury recovery process.
Iliff et al. [44]	Mice	To evaluate if AQP4 gene deletion is associated to the buildup of phosphorylated tau protein after TBI.	‘Hit & Run” traumatic brain injury model to induce moderate TBI by using a pneumatic-controlled, cortical impact device (using a strike depth of 10 mm and 0.1 time of impact with an impact velocity of 5.2 m/s).	Twenty-eight days after TBI, in wild-type mice mild P-tau immunoreactivity was evident in the cortex surrounding the traumatic lesion, while in Aqp4-/- mice P-tau immunoreactivity was pronounced. Behavioral test used: - Open field test: to investigate spontaneous locomotor activity; no significant difference was observed in wild-type mice after TBI while in Aqp4-/- mice, open field test performance was impaired after TBI. - Rotarod test: to measure the latency before falling from the rod; TBI did not significantly impair function in wild-type mice, in Aqp4-/- mice performance was significantly impaired after injury. - Novel-object recognition to evaluate recognition memory, and Barnes maze test to measure spatial learning and memory; TBI impaired cognitive function both in wild-type and Aqp4-/- mice. Aqp4 gene deletion cause glymphatic pathway dysfunction and promotes post-traumatic neuroinflammation, exacerbating cognitive deficits after TBI.
Li et al. [45]	Mice	To investigate mild TBI effect on the glymphatic system using contrast-enhanced MRI.	mTBI induced before exposing the skull, by dropping a cylindrical column of segmented brass (450 g) from a height of 1 m.	mTBI determines lower infusion and clearance rate of contrast agent in cortex, hippocampus and thalamus than in hypothalamus, olfactory bulb and cerebellum. No behavioral/neurological test were performed.
Liu et al. [46]	Mice	To study the role of AQP4 in progression of TBI in knockout mice.	Cortical impact injury (CCI). TBI severity not specified.	AQP4 absence improve the symptoms of TBI, protect the integrity of the brain-blood barrier (BBB), promote the clearance of brain amyloid beta, and inhibit the inflammatory response in the brain. Behavioral test used: - Morris water maze test to investigate the alteration of the learning ability and memory of the AQP4−/− mice; AQP4 knockout promoted the recovery of learning ability of TBI mice. AQP4 deficiency reduce brain edema improving TBI-induced neurological outcomes.
Liu et al. [47]	Mice	To explore the role of AQP4 using MRI after subarachnoid hemorrhage (SAH).	SAH established using endovascular perforation method (anesthetized rat in which a nylon suture was inserted through the right internal carotid artery to perforate the junction of the middle and anterior cerebral arteries).	After SAH, the diffusion of Gd-DTPA injected into the cisterna magna was markedly blocked in Aqp4−/−rats, which showed more severe brain edema aggravating neurological deficits. Neurological functions tests used: spontaneous activity, symmetry of the limb movement, forepaw outstretching, climbing, body proprioception, and whisker stimulation response. AQP4 played important roles in early brain injury following SAH.
Zhang et al. [48]	Mice	To investigate the effects of Omega-3 in promoting waste clearance thorough the glymphatic system in induced TBI model.	TBI induced by CCI apparatus. After making an incision to expose the skull; a hole of 3.5 mm in diameter was drilled on the right hemisphere 2.0 mm lateral to the midline and 2.0 mm from Bregma; impact applicator was applied at a depth of 2 mm at 4.5 m/s for 200 ms.	Administering fish oil (rich in Omega-3) for a period of two months before determining a TBI significantly improves neuronal function in mouse models, favors the clearance of radiolabeled tracers and prevents the accumulation of Aß by partially restoring the expression and depolarization of AQP4, impaired by TBI. Neurological functions tests used: - Modified Neurological Severity Score (mNSS) to assess the reflex, balance, sensory and motor functions; TBI group showed significant neurological impairment following CCI (increased mNSS). Fish oil supplementation for 2 months prior to TBI induction significantly reduced mNSS. - Rota-rod test to investigate motor coordination; TBI group showed significant neurological impairment following CCI (reduced fall latency). Mice supplemented with fish oil showed significant improved performance after induction of TBI.
Plog et al. [49]	Mice	To alter the glymphatic function through four different mechanisms: the deletion of AQP4; the administration of acetazolamide (which inhibits the production of cerebrospinal fluid at the level of the choroid plexuses); the execution of a cysternotomy of the cisterna magna (which promotes cerebrospinal fluid drainage and reduces glymphatic system function); and sleep deprivation (which has been shown to reduce glymphatic efflux).	“Hit & Run” TBI model: a CCI device was employed using an impactor velocity of 4.7 m/s, an impact depth of 10 mm, and impact duration of 100 ms. The location of the impact was the point 4.5 mm lateral to midline and 4.5 mm posterior to the left orbit.	By cortical administration of a protein tracer, it was observed that these four mechanisms cause a reduction in clearance compared to the control group. The clearance of specific markers for TBI such as S100b, GFAP, and NSE was evaluated, showing that in wild-type mice there was a significant increase in serum levels of these markers after TBI, while, in AQP4 knockout mice and mice undergoing cysternotomy, acetazolamide administration, and sleep deprivation, the serum levels were similar to those of wild-type mice not subjected to the trauma. No behavioral/neurological test were performed.
Opel et al. [50]	Humans	To evaluate the relationship between TBI and enlargement of the perivascular spaces (ePVS) using brain MRI and overnight polysomnography.	TBI severity not specified.	The enlargement of the perivascular spaces is independently related to a reduction in sleep time. However, this correlation was shown to be more significant in subjects who had undergone a TBI than in the group that did have TBI history. The results suggest that ePVS may be modulated by sleep and TBI and that there may be a causative relationship between loss of glymphatic function and dilation of the perivascular space.
Piantino et al. [51]	Humans	To determine the effect of mTBI on visible perivascular spaces using MRI in military veterans.	Military blast mTBI.	A significant correlation was found between mild TBI and PVS burden, which suggest waste clearance dysfunction.
Ren et al. [52]	Mice	To characterize changes in aquaporin-4 (AQP4) expression and localization after mild and moderate TBI.	“Hit & Run” TBI model using a strike depth of 10 mm, and 0.1 s of contact time, with a velocity of 4.8 m/s for mild and 5.2 m/s for moderate TBI.	After TBI, AQP4 expression was increased, with a loss of polarized localization at the end foot of reactive astrocytes, suggesting that changes in AQP4 may not contribute to cerebral edema formation, but may represent a compensatory mechanism to facilitate its resolution. Neurological functions tests used: - Gross neuroscore to evaluate changes in physiologic state, motor function, and alertness; mild TBI animals exhibited neuroscore deficits at 3 and 24 h after TBI, resolving before 3 days post injury; moderate TBI animals were significantly impaired compared with mild TBI animals, but global neuroscore normalized within 7 days post injury. - Rotarod test to evaluate motor performance; mice subjected to both mild and moderate TBI exhibited mild, yet significant impairment in rotarod performance throughout the 4 weeks post injury. - Open field test to evaluate general motor function and anxiety; no significant effect of TBI was found. - Novel object recognition test was used to monitor cognitive function; mild TBI animals did not exhibit any deficit, moderate TBI animals demonstrated a significant decline in test performance. - Barnes maze test to evaluate the hippocampal-dependent cognitive function; mild TBI animals exhibited no deficit, moderate TBI animals demonstrated a significant cognitive deficit compared with the control group.
Olczak et al. [53]	Humans	To check the relevance of MAPT examination for forensic purposes in head injury death.	Severe head injury.	MAPT serum and cerebrospinal fluid levels should be considered a TBI marker in postmortem examination.
Lindblad et al. [54]	Humans	To assess if and how clearance of S100 B and NSE (brain enriched proteins) from brain to blood is affected by BBB disruption in severe TBI patients.	Severe TBI.	Intracranial NSE accumulates and is cleared through a route other than the BBB, which may be the glymphatic system.

## Data Availability

Not applicable.

## References

[1] Iliff J.J., Wang M., Liao Y., Plogg B.A., Peng W., Gundersen G.A., Benveniste H., Vates G.E., Deane R., Goldman S.A. (2012). A paravascular pathway facilitates CSF flow through the brain parenchyma and the clearance of interstitial solutes, including amyloid β. Sci. Transl. Med..

[2] Silva I., Silva J., Ferreira R., Trigo D. (2021). Glymphatic system, AQP4, and their implications in Alzheimer’s disease. Neurol. Res. Pract..

[3] Peng W., Achariyar T.M., Li B., Liao Y., Mestre H., Hitomi E., Regan S., Kasper T., Peng S., Ding F. (2016). Suppression of glymphatic fluid transport in a mouse model of Alzheimer’s disease. Neurobiol. Dis..

[4] Zou W., Pu T., Feng W., Lu M., Zheng Y., Du R., Xiao M., Hu G. (2019). Blocking meningeal lymphatic drainage aggravates Parkinson’s disease-like pathology in mice overexpressing mutated α-synuclein. Transl. Neurodegener..

[5] Nedergaard M., Goldman S.A. (2020). Glymphatic failure as a final common pathway to dementia. Science.

[6] Christensen J., Yamakawa G.R., Shultz S.R., Mychasiuk R. (2021). Is the glymphatic system the missing link between sleep impairments and neurological disorders? Examining the implications and uncertainties. Prog. Neurobiol..

[7] Wostyn P., de Groot V., van Dam D., Audenaert K., de Deyn P.P., Killer H.E. (2016). The Glymphatic System: A New Player in Ocular Diseases?. Investig. Ophthalmol. Vis. Sci..

[8] Zamani A., Walker A.K., Rollo B., Ayers K.L., Farah R., O’Brien T.J., Wright D.K. (2022). Impaired glymphatic function in the early stages of disease in a TDP-43 mouse model of amyotrophic lateral sclerosis. Transl. Neurodegener..

[9] Sullan M.J., Asken B.M., Jaffee M.S., DeKosky S.T., Bauer R.M. (2018). Glymphatic system disruption as a mediator of brain trauma and chronic traumatic encephalopathy. Neurosci. Biobehav. Rev..

[10] Piantino J., Lim M.M., Newgard C.D., Iliff J. (2019). Linking Traumatic Brain Injury, Sleep Disruption and Post-Traumatic Headache: A Potential Role for Glymphatic Pathway Dysfunction. Curr. Pain Headache Rep..

[11] Iliff J.J., Goldman S.A., Nedergaard M. (2015). Implications of the discovery of brain lymphatic pathways. Lancet Neurol..

[12] Capizzi A., Woo J., Verduzco-Gutierrez M. (2020). Traumatic Brain Injury: An Overview of Epidemiology, Pathophysiology, and Medical Management. Med. Clin..

[13] Betrus C., Kreipke C.W. (2013). Historical perspectives in understanding traumatic brain injury and in situating disruption in CBF in the pathotrajectory of head trauma. Cerebral Blood Flow, Metabolism and Head Trauma: The Pathotrajectory of Traumatic Brain Injury.

[14] Wang G., Zhang Y.P., Gao Z., Shields L.B.E., Li F., Chu T., Lv H., Moriarty T., Xu X.M., Yang X. (2018). Pathophysiological and behavioral deficits in developing mice following rotational acceleration-deceleration traumatic brain injury. DMM Dis. Models Mech..

[15] Palmieri M., Frati A., Santoro A., Frati P., Fineschi V., Pesce A. (2021). Diffuse Axonal Injury: Clinical Prognostic Factors, Molecular Experimental Models and the Impact of the Trauma Related Oxidative Stress. An Extensive Review Concerning Milestones and Advances. Int. J. Mol. Sci..

[16] Maiese A., Iannaccone F., Scatena A., Del Fante Z., Oliva A., Frati P., Fineschi V. (2021). Pediatric Abusive Head Trauma: A Systematic Review. Diagnostics.

[17] Martinez A.P., Scherer M.J., Tozser T. (2018). Traumatic Brain Injury (TBI) in School-Based Populations: Common Sequelae and Assistive Technology Interventions. Adv. Neurodev. Disord..

[18] Centers for Disease Control and Prevention National Center for Health Statistics: Mortality Data on CDC WONDER. https://wonder.cdc.gov/mcd.html.

[19] Alkhier Ahmed A., Najb Almulla N., Ali Ahli M., Abdelrahman Alzarouni A. (2021). Pituitary dysfunction following a traumatic brain injury (TBI) at the desk of a General Practitioner. World Fam. Med..

[20] Laeke T., Tirsit A., Kassahun A., Sahlu A., Debebe T., Yesehak B., Masresha S., Deyassa N., Moen B.E., Lund-Johansen M. (2021). Prospective Study of Surgery for Traumatic Brain Injury in Addis Ababa, Ethiopia: Trauma Causes, Injury Types, and Clinical Presentation. World Neurosurg..

[21] Iarussi F., Cipolloni L., Bertozzi G., Sasso L., Ferrara M., Salerno M., Rubino G.T.R., Maglietta F., Dinisi A., Albano D. (2020). Dog-bite-related attacks: A new forensic approach. Forensic Sci. Int..

[22] Balakrishnan B., Rus R.M., Chan K.H., Martin A.G., Awang M.S. (2019). Prevalence of Postconcussion Syndrome after Mild Traumatic Brain Injury in Young Adults from a Single Neurosurgical Center in East Coast of Malaysia. Asian J. Neurosurg..

[23] Langlois J., Rutland-Brown W., Wald M.M. (2006). The epidemiology and impact of traumatic brain injury: A brief overview. J. Head Trauma Rehabil..

[24] Ghajar J. (2000). Traumatic brain injury. Lancet.

[25] Neri M., Büttner A., Fineschi V. (2017). Brain Injury due to Mechanical Trauma and Ischemic-Hypoxic Insult: Biomarkers of Brain Injury and Oxidative Stress. Oxid. Med. Cell. Longev..

[26] Ng S.Y., Lee A.Y.W. (2019). Traumatic Brain Injuries: Pathophysiology and Potential Therapeutic Targets. Front. Cell. Neurosci..

[27] Frati A., Cerretani D., Fiaschi A.I., Frati P., Gatto V., La Russa R., Pesce A., Pinchi E., Santurro A., Fraschetti F. (2017). Diffuse Axonal Injury and Oxidative Stress: A Comprehensive Review. Int. J. Mol. Sci..

[28] Kaur P., Sharma S. (2017). Recent Advances in Pathophysiology of Traumatic Brain Injury. Curr. Neuropharmacol..

[29] Dell’Aquila M., Maiese A., De Matteis A., Viola R.V., Arcangeli M., La Russa R., Fineschi V. (2021). Traumatic brain injury: Estimate of the age of the injury based on neuroinflammation, endothelial activation markers and adhesion molecules. Histol. Histopathol..

[30] La Russa R., Maiese A., Cipolloni L., Di Fazio N., Delogu G., De Matteis A., Del Fante Z., Manetti F., Frati P., Fineschi V. (2022). Diagnostic assessment of traumatic brain injury by vacuum extraction in newborns: Overview on forensic perspectives and proposal of operating procedures. Front. Biosci..

[31] Cafarelli F.P., Grilli G., Zizzo G., Bertozzi G., Giuliani N., Mahakkanukrauh P., Pinto A., Guglielmi G. (2019). Postmortem Imaging: An Update. Semin. Ultrasound CT MRI.

[32] Ferrara M., Sessa F., Rendine M., Spagnolo L., De Simone S., Riezzo I., Ricci P., Pascale N., Salerno M., Bertozzi G. (2019). A multidisciplinary approach is mandatory to solve complex crimes: A case report. Egypt. J. Forensic Sci..

[33] Aromatario M., Torsello A., D’errico S., Bertozzi G., Sessa F., Cipolloni L., Baldari B. (2021). Traumatic Epidural and Subdural Hematoma: Epidemiology, Outcome, and Dating. Medicina.

[34] Zanza C., Piccolella F., Racca F., Romenskaya T., Longhitano Y., Franceschi F., Savioli G., Bertozzi G., De Simone S., Cipolloni L. (2022). Ketamine in Acute Brain Injury: Current Opinion Following Cerebral Circulation and Electrical Activity. Healthcare.

[35] La Russa R., Maiese A., Di Fazio N., Morano A., Di Bonaventura C., De Matteis A., Fazio V., Frati P., Fineschi V. (2020). Post-Traumatic Meningitis Is a Diagnostic Challenging Time: A Systematic Review Focusing on Clinical and Pathological Features. Int. J. Mol. Sci..

[36] Neri M., Frati A., Turillazzi E., Cantatore S., Cipolloni L., Di Paolo M., Frati P., La Russa R., Maiese A., Scopetti M. (2018). Immunohistochemical Evaluation of Aquaporin-4 and its Correlation with CD68, IBA-1, HIF-1α, GFAP, and CD15 Expressions in Fatal Traumatic Brain Injury. Int. J. Mol. Sci..

[37] Fineschi V., Viola R.V., La Russa R., Santurro A., Frati P. (2017). A Controversial Medicolegal Issue: Timing the Onset of Perinatal Hypoxic-Ischemic Brain Injury. Mediat. Inflamm..

[38] Turillazzi E., Karch S.B., Neri M., Pomara C., Riezzo I., Fineschi V. (2008). Confocal laser scanning microscopy. Using new technology to answer old questions in forensic investigations. Int. J. Leg. Med..

[39] Pinchi E., Frati A., Cipolloni L., Aromatario M., Gatto V., La Russa R., Pesce A., Santurro A., Fraschetti F., Frati P. (2018). Clinical-pathological study on β-APP, IL-1β, GFAP, NFL, Spectrin II, 8OHdG, TUNEL, miR-21, miR-16, miR-92 expressions to verify DAI-diagnosis, grade and prognosis. Sci. Rep..

[40] Page M.J., McKenzie J.E., Bossuyt P.M., Boutron I., Hoffmann T.C., Mulrow C.D., Shamseer L., Tetzlaff J.M., Akl E.A., Brennan S.E. (2021). The PRISMA 2020 statement: An updated guideline for reporting systematic reviews. Syst. Rev..

[41] Viera A.J., Garrett J.M. (2005). Understanding interobserver agreement: The kappa statistic. Fam. Med..

[42] Falavigna A., Blauth M., Kates S.L. (2017). Critical review of a scientific manuscript: A practical guide for reviewers. J. Neurosurg..

[43] Christensen J., Wright D.K., Yamakawa G.R., Shultz S.R., Mychasiuk R. (2020). Repetitive Mild Traumatic Brain Injury Alters Glymphatic Clearance Rates in Limbic Structures of Adolescent Female Rats. Sci. Rep..

[44] Iliff J.J., Chen M.J., Plog B.A., Zeppenfeld D.M., Soltero M., Yang L., Singh I., Deane R., Nedergaard M. (2014). Impairment of glymphatic pathway function promotes tau pathology after traumatic brain injury. J. Neurosci..

[45] Li L., Chopp M., Ding G., Davoodi-Bojd E., Zhang L., Li Q., Zhang Y., Xiong Y., Jiang Q. (2020). MRI detection of impairment of glymphatic function in rat after mild traumatic brain injury. Brain Res..

[46] Liu X., Xie Y., Wan X., Wu J., Fan Z., Yang L. (2021). Protective Effects of Aquaporin-4 Deficiency on Longer-term Neurological Outcomes in a Mouse Model. Neurochem. Res..

[47] Liu E., Sun L., Zhang Y., Wang A., Yan J. (2020). Aquaporin4 Knockout Aggravates Early Brain Injury Following Subarachnoid Hemorrhage Through Impairment of the Glymphatic System in Rat Brain. Acta Neurochir. Suppl..

[48] Zhang E., Wan X., Yang L., Wang D., Chen Z., Chen Y., Liu M., Zhang G., Wu J., Han H. (2020). Omega-3 Polyunsaturated Fatty Acids Alleviate Traumatic Brain Injury by Regulating the Glymphatic Pathway in Mice. Front. Neurol..

[49] Plog B.A., Dashnaw M.L., Hitomi E., Peng W., Liao Y., Lou N., Deane R., Nedergaard M. (2015). Biomarkers of Traumatic Injury Are Transported from Brain to Blood via the Glymphatic System. J. Neurosci..

[50] Opel R.A., Christy A., Boespflug E.L., Weymann K.B., Case B., Pollock J.M., Silbert L.C., Lim M.M. (2019). Effects of traumatic brain injury on sleep and enlarged perivascular spaces. J. Cereb. Blood Flow Metab..

[51] Piantino J., Schwartz D.L., Luther M., Newgard C., Silbert L., Raskind M., Pagulayan K., Kleinhans N., Iliff J., Peskind E. (2021). Link between Mild Traumatic Brain Injury, Poor Sleep, and Magnetic Resonance Imaging: Visible Perivascular Spaces in Veterans. J. Neurotrauma.

[52] Ren Z., Iliff J.J., Yang L., Yang J., Chen X., Chen M.J., Giese R.N., Wang B., Shi X., Nedergaard M. (2013). “Hit & Run” model of closed-skull traumatic brain injury (TBI) reveals complex patterns of post-traumatic AQP4 dysregulation. J. Cereb. Blood Flow Metab..

[53] Olczak M., Niderla-Bielińska J., Kwiatkowska M., Samojłowicz D., Tarka S., Wierzba-Bobrowicz T. (2017). Tau protein (MAPT) as a possible biochemical marker of traumatic brain injury in postmortem examination. Forensic Sci. Int..

[54] Lindblad C., Nelson D.W., Zeiler F.A., Ercole A., Ghatan P.H., Von Horn H., Risling M., Svensson M., Agoston D.V., Bellander B.M. (2020). Influence of Blood–Brain Barrier Integrity on Brain Protein Biomarker Clearance in Severe Traumatic Brain Injury: A Longitudinal Prospective Study. J. Neurotrauma.

[55] Plog B.A., Nedergaard M. (2018). The Glymphatic System in Central Nervous System Health and Disease: Past, Present, and Future. Annu. Rev. Pathol..

[56] Mestre H., Mori Y., Nedergaard M. (2020). The Brain’s Glymphatic System: Current Controversies. Trends Neurosci..

[57] Jessen N.A., Munk A.S.F., Lundgaard I., Nedergaard M. (2015). The Glymphatic System—A Beginner’s Guide. Neurochem. Res..

[58] Wu Z., Wang Z.H., Liu X., Zhang Z., Gu X., Yu S.P., Keene C.D., Cheng L., Ye K. (2020). Traumatic brain injury triggers APP and Tau cleavage by delta-secretase, mediating Alzheimer’s disease pathology. Prog. Neurobiol..

[59] Graham N.S.N., Sharp D.J. (2019). Understanding neurodegeneration after traumatic brain injury: From mechanisms to clinical trials in dementia. J. Neurol. Neurosurg. Psychiatry.

[60] Ferrara M., Bertozzi G., Zanza C., Longhitano Y., Piccolella F., Lauritano C.E., Volonnino G., Manetti A.C., Maiese A., La Russa R. (2022). Traumatic Brain Injury and Gut Brain Axis: The Disruption of an Alliance. Rev. Recent Clin. Trials.

[61] Bertozzi G., Maglietta F., Sessa F., Scoto E., Cipolloni L., Di Mizio G., Salerno M., Pomara C. (2020). Traumatic Brain Injury: A Forensic Approach: A Literature Review. Curr. Neuropharmacol..

[62] Tomura S., Nawashiro H., Otani N., Uozumi Y., Toyooka T., Ohsumi A., Shima K. (2011). Effect of decompressive craniectomy on aquaporin-4 expression after lateral fluid percussion injury in rats. J. Neurotrauma.

[63] Planel E., Bretteville A., Liu L., Virag L., Du A.L., Yu W.H., Dickson D.W., Whittington R.A., Duff K.E. (2009). Acceleration and persistence of neurofibrillary pathology in a mouse model of tauopathy following anesthesia. FASEB J..

